# Race, Ethnicity and Teaching the ‘Mental State Examination’

**DOI:** 10.1192/bjo.2026.11341

**Published:** 2026-06-30

**Authors:** Rhian Bradley, Ifeoluwa Oladejo

**Affiliations:** 1 Kent and Medway Mental Health NHS Trust, Dartford, United Kingdom; 2 Dartford and Gravesham NHS Trust, Dartford, United Kingdom

## Abstract

**Aims:**

During a routine presentation of a ‘mental state examination’ (MSE) within clinical supervision, we noted a hesitancy to describe a patient’s appearance with regards to their race and ethnicity, in a manner routinely taught during training. Our aim was to critically engage with relevant literature to inform our understanding of whether teaching the routine inclusion of race and ethnic descriptors within the MSE should be challenged.

**Methods:**

We conducted a review of relevant literature. Six articles were included (full references available).

**Results:**

Limitations within diagnostic formulation: Race, associated with physical characteristics, and ethnicity, associated with descent and cultural factors, are poorly defined, Racial categories do not robustly represent biological predisposition to mental illness. Ethnicity might be considered a ‘shortcut’ to understanding an individual’s sociocultural predisposition to mental illness, however this is shaped by broader societal influences across the lifespan.

Risks of stereotyping: Despite a decline in stereotyping of patients by psychiatrists, racial stereotyping, whether arising from conscious or unconscious biases, remains an issue. Such stereotyping can lead to inaccurate assumptions which may contribute to disparities in diagnosis and healthcare delivery.

Impact of racial/ethnic mis-assignment: Visual assessments of race/ethnicity, based on offhand observations, risk mis-assignment. There is evidence from non-clinical settings that this has a negative impact on sense of identity and mental health. In a specialty where patients often have a complex sense of their own identity, this may cause additional unintended harm. A positive view of one’s ‘self’ is important to recovery; conversely invalidation of a patient’s ‘knowledge of self’ may contribute to epistemic injustice. Being mis-labelled by professionals may also reinforce mistrust of mental health services by some ethnic minorities, contributing to perpetuation of inequalities in accessing care.

Risks of ‘colour blind’ practice: Recognising and understanding an individual’s ethnicity may validate a patient’s sense of identity and is vital for providing holistic care. Recording, recognising and researching race and ethnicity is crucial to better understand and address health disparities within mental health.

**Conclusion:**

Understanding patients’ ethnic and cultural identities is crucial to appreciate and respond to their mental health difficulties. However, we challenge teaching the routine inclusion of race and ethnic descriptors within the ‘mental state examination’. It has little value in supporting formulation and treatment planning, and may contribute towards damaging stereotyping and mis-assignment. Teaching psychiatric trainees to engage in conversations around patients’ heritage and to incorporate relevant aspects within their formulation and treatment is crucial.

